# Supplementary material for: MetaRibo-Seq measures translation in microbiomes
Source: Nat Commun. 2020 Jun 29;11:3268. doi: 10.1038/s41467-020-17081-z (PMC7324362; doi:10.1038/s41467-020-17081-z)
Supplement: Supplementary file 10 — Supplementary Data 7 [file 41467_2020_17081_MOESM10_ESM.zip › File2/Confidence_VeryHigh_Taxonomy/136867_out.krona.html]

Javascript must be enabled to view this page.

members
magnitude
magnitudeUnassigned
count
unassigned
taxon
rank

136867\_out

23

1
superkingdom
23

SRS019068\_contig\_number\_32855
2

5
1239
phylum

class
186801
5

186802
order
5

424536
family
2

2
270497
genus

2

SRS013098\_contig\_number\_38375SRS077392\_contig\_number\_11634
1946292
species

2
186803
family

1432051
genus
2

2

SRS049773\_contig\_number\_contig-100\_30371.128777SRS104400\_contig\_number\_contig-100\_14189.93875
species
2292348

1
541000
family

1263
genus
1


SRS1041145\_contig\_number\_19141
1262957
species
1

17
phylum
1224

17
28211
class

17
order
204441

17
41295
family

17
genus
191


SRS016095\_contig\_number\_182SRS017701\_contig\_number\_12724SRS017821\_contig\_number\_contig-100\_3805.148619SRS018836\_contig\_number\_21396SRS024331\_contig\_number\_25010SRS043768\_contig\_number\_15533SRS050422\_contig\_number\_36035SRS053649\_contig\_number\_contig-100\_291.141520SRS076804\_contig\_number\_21154SRS076929\_contig\_number\_contig-100\_1535.122610SRS1041132\_contig\_number\_1741SRS1041145\_contig\_number\_10654SRS104311\_contig\_number\_16861SRS143523\_contig\_number\_16405SRS143895\_contig\_number\_21731SRS144135\_contig\_number\_857SRS148874\_contig\_number\_14226
species
1896972
17
